# Contrast Echocardiography: Unveiling Eccentric Mitral Regurgitation

**DOI:** 10.1016/j.case.2023.11.001

**Published:** 2023-12-21

**Authors:** Sam Ostad Karampour, Matthew Church, Jonathan Choy

**Affiliations:** Mazankowski Alberta Heart Institute, University of Alberta, Edmonton, Alberta, Canada

**Keywords:** Contrast transthoracic echocardiography, Mitral valve prolapse, Eccentric mitral regurgitation, Ultrasound enhancing agent

## Abstract

• Leaflet prolapse, flail, and perforation often result in eccentric MR.
• Eccentric jets appear smaller than central jets of similar severity on color Doppler.
• The use of UEAs is helpful in better visualizing eccentric MR jets on TTE.

## Graphical abstract

**Figure:**
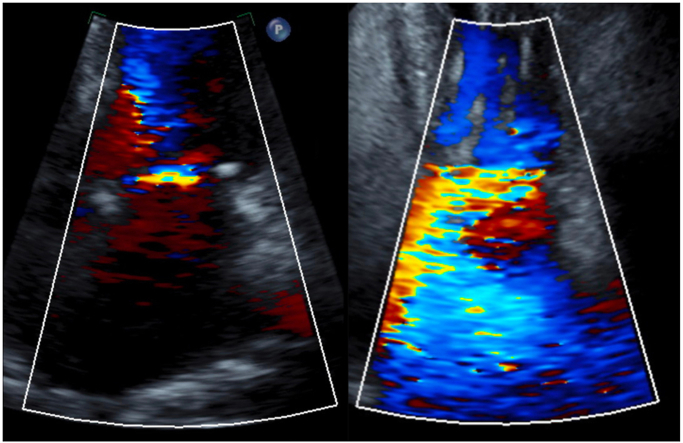

## Introduction

Mitral regurgitation (MR) is the most common valvular abnormality worldwide.^1^ Regurgitant jets are often eccentric in the presence of regional wall motion abnormalities and when the underlying pathology is leaflet prolapse, flail, or perforation. Eccentric jets hug the left atrial wall because of the Coandă effect, the tendency of a jet to follow the contour of a convex surface.^2^ As a result, the severity of an eccentric jet appears smaller on color flow Doppler in comparison with central jets of the same hemodynamic severity.^3^ This may lead to overlooking or grossly underestimating MR severity on two-dimensional (2D) transthoracic echocardiography (TTE). Here we present a patient with mitral valve (MV) prolapse with severe eccentric regurgitation on TTE that was initially not well visualized on nonenhanced imaging but highlighted after administration of an ultrasound enhancing agent (UEA).

## Case Presentation

**Figure 1:**
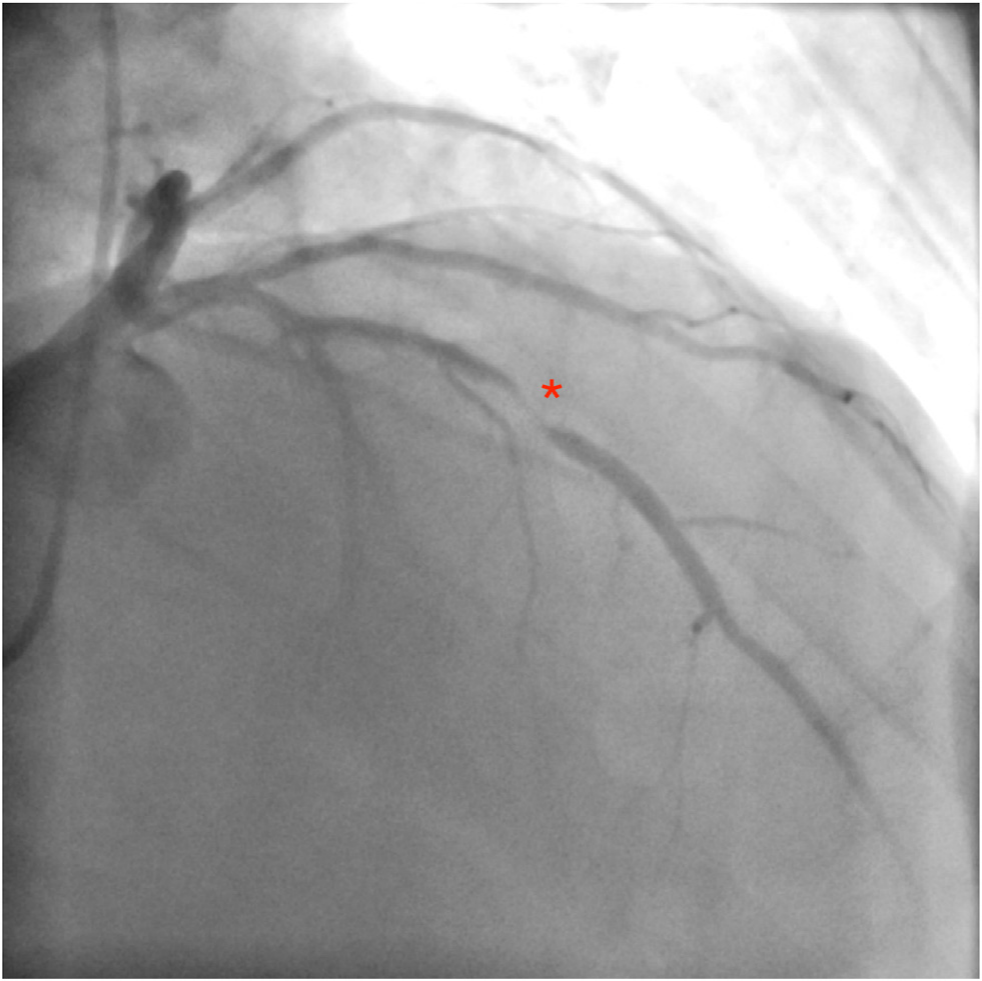
Coronary angiogram (right anterior oblique view) demonstrates significant mid left anterior descending coronary artery stenosis (*asterisk*).

A 75-year-old man with a history of osteoarthritis contacted emergency medical services because of acute onset of retrosternal chest pain. Electrocardiography demonstrated anterior ST-segment elevation infarction, for which the patient was administered dual-antiplatelet therapy and fibrinolysis before transfer to the hospital. On arrival to the coronary care unit, the patient had clinical reperfusion, evident by resolution of chest pain and ST-segment elevation on electrocardiography. On history, the patient endorsed a 3-day history of exertional chest pain on a background of chronic mild exertional dyspnea. They denied other cardiac symptoms, including orthopnea, paroxysmal nocturnal dyspnea, pedal edema, palpitations, and presyncope. Cardiac examination was remarkable for a grade III/VI holosystolic murmur, heard loudest over the apex. Less than 24 hours after presentation, the patient underwent coronary angiography demonstrating an 80% mid left anterior descending coronary artery culprit lesion, which was successfully treated with a single drug-eluting stent (Figure 1). Left ventricular (LV) end-diastolic pressure was normal at 5 mm Hg.

**Figure 2:**
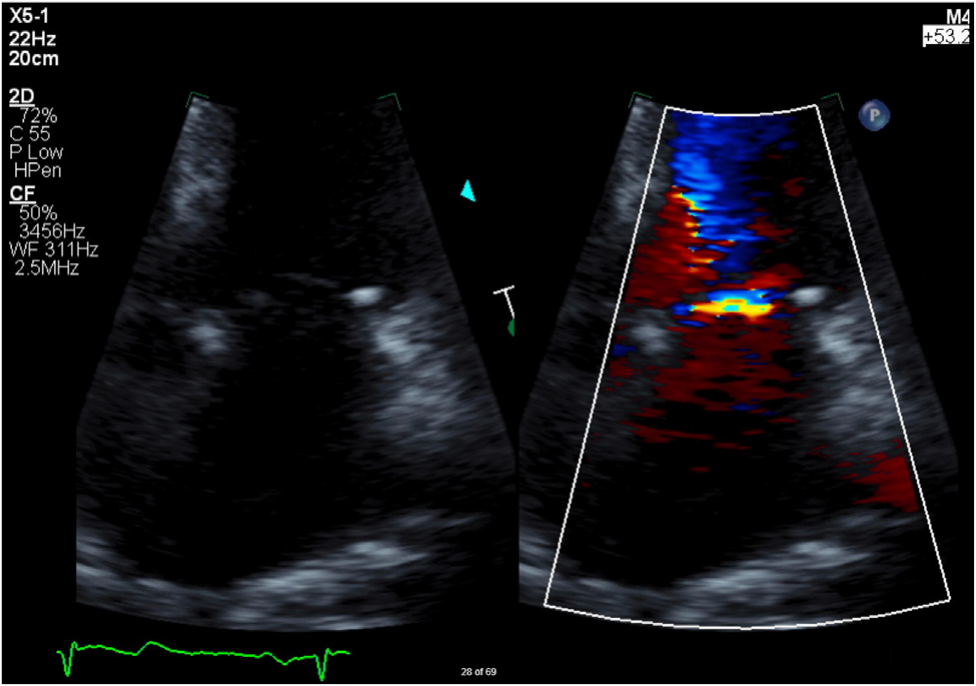
Nonenhanced 2D TTE, zoomed-in apical four-chamber view, without (*left*) and with (*right*) color flow Doppler in systolic phase, demonstrates a distinct horizontal line of color signal across the mitral annular surface of the left atrium, without evidence of any further regurgitant jet.

**Figure 3:**
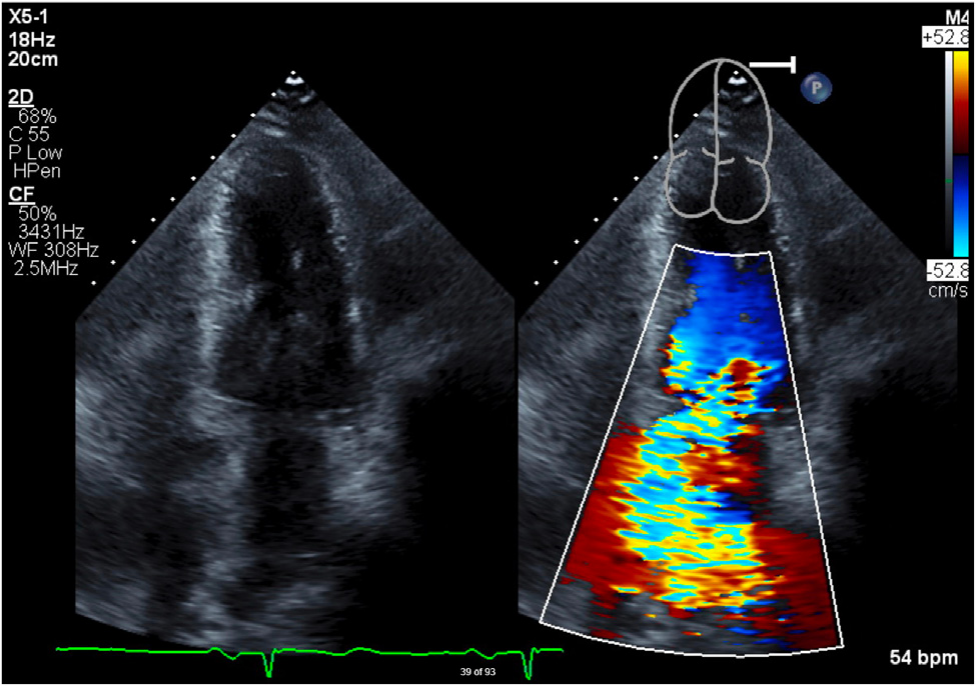
Two-dimensional TTE, apical four-chamber view, without (*left*) and with (*right*) color flow Doppler in systolic phase after administration of a UEA, demonstrates a large eccentric anteriorly directed MR jet.

Forty-eight hours after admission, TTE was performed to assess LV ejection fraction (LVEF) and for the presence of apical thrombus. TTE demonstrated anteroapical wall motion abnormalities with preserved LVEF of 59% by the biplane method of disks (Video 1). LV end-systolic diameter was borderline normal at 3.8 cm, with a severely dilated left atrium at 70 mL/m^2^. Nonenhanced high–mechanical index (0.9-1.2) imaging revealed a trileaflet sclerotic aortic valve with no valvular stenosis or regurgitation. There was mild mitral annular calcification with a distinct horizontal line of color signal across the annular surface of the atrium using color flow Doppler at higher Nyquist limits (53-62 cm/sec); however, there was no prominent regurgitant jet on apical windows to suggest significant MR (Figure 2, Video 2, Video 3, Video 4). Of note, mitral peak E-wave velocity was elevated at 119 cm/sec^2^. Additionally, there was normal functioning pulmonary valve with mild tricuspid regurgitation (TR). A UEA (activated lipid microspheres) was injected intravenously to assess for apical thrombus. Low–mechanical index (<0.2) contrast echocardiography did not reveal a thrombus; however, high–mechanical index (1.0) enhanced color flow Doppler did highlight a large eccentric anterior mitral regurgitant jet covering >50% of the left atrium, suggestive of more severe MR at a Nyquist limit of 53 cm/sec (Figure 3, Video 5). Additionally, contrast-enhanced imaging demonstrated moderately dilated LV end-diastolic (188 mL) and end-systolic (76 mL) volumes. Given findings on TTE suggestive of severe MR, in addition to borderline LVEF and LV end-systolic diameter meeting guideline indications for intervention, transesophageal echocardiogram (TEE) was performed to confirm MR severity.

**Figure 4:**
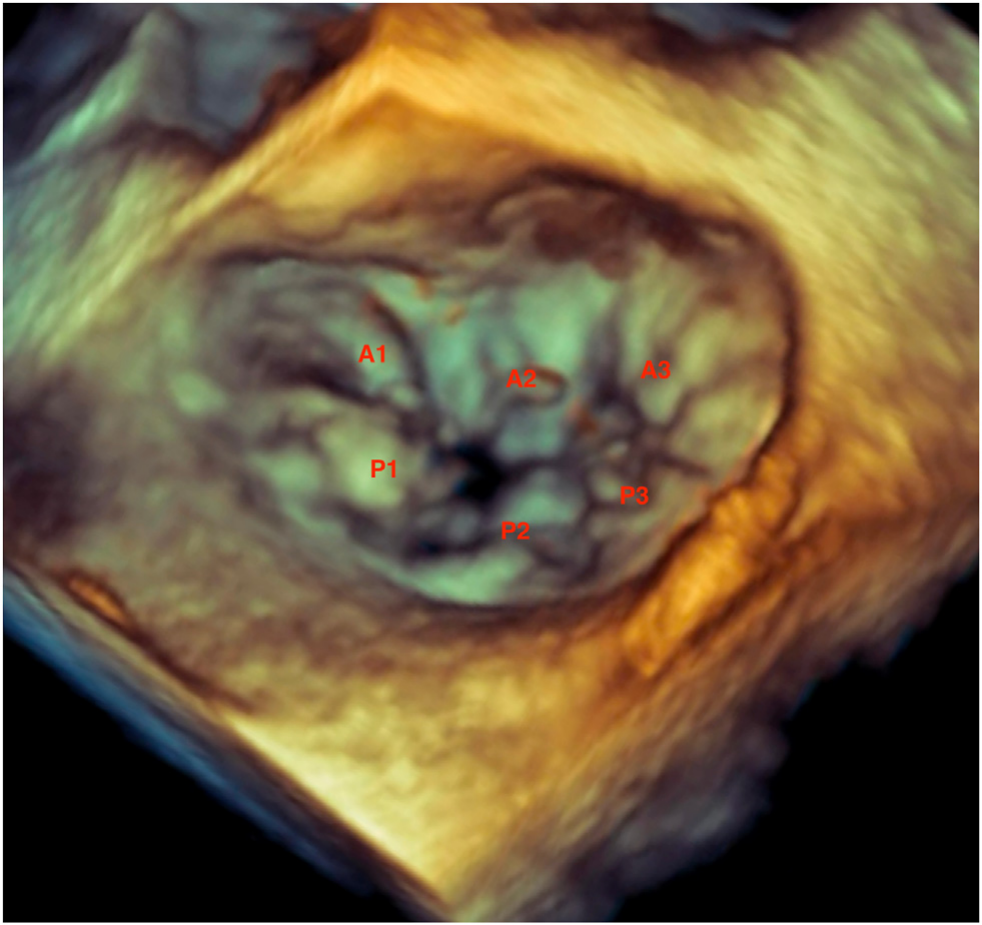
Three-dimensional TEE, midesophageal position, volume-rendered display, surgeon’s view of the MV, systolic phase, demonstrates the flail P1 scallop and small flail segment of the lateral commissure.

**Figure 5:**
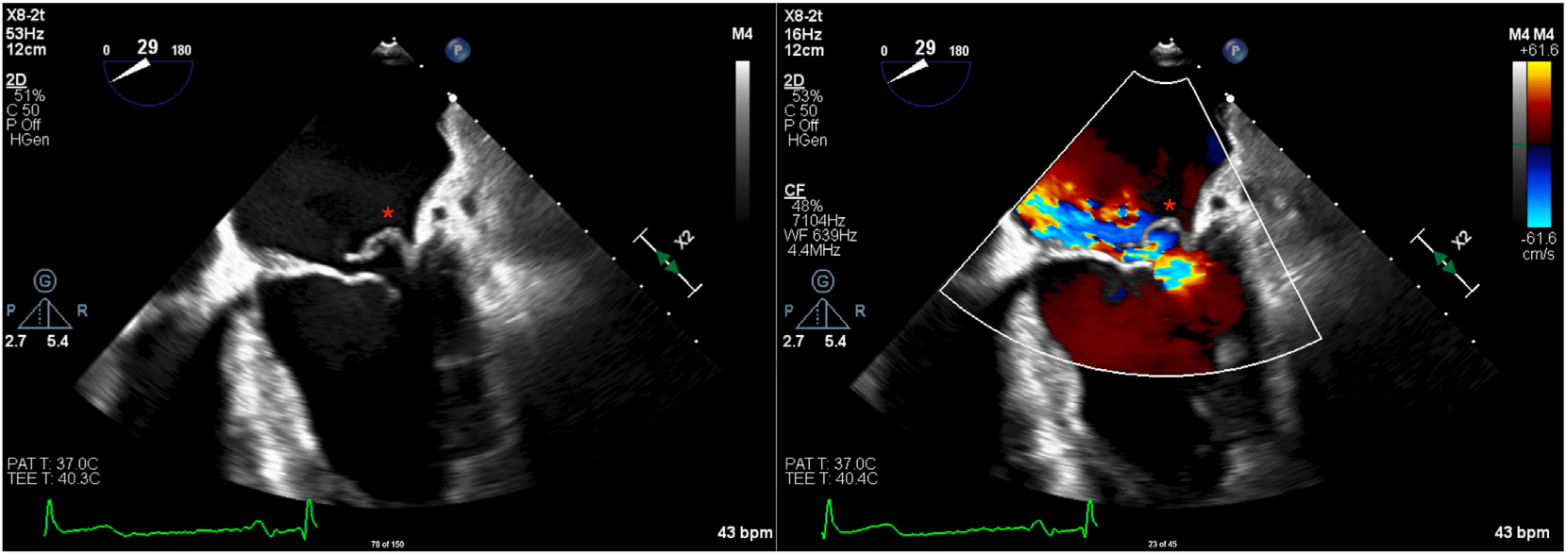
Two-dimensional TEE, midesophageal four-chamber (29°) view, systolic phase without (*left*) and with (*right*) color flow Doppler, demonstrates the posterior flail leaflet (*asterisk*) and very eccentric, anteriorly directed MR jet.

**Figure 6:**
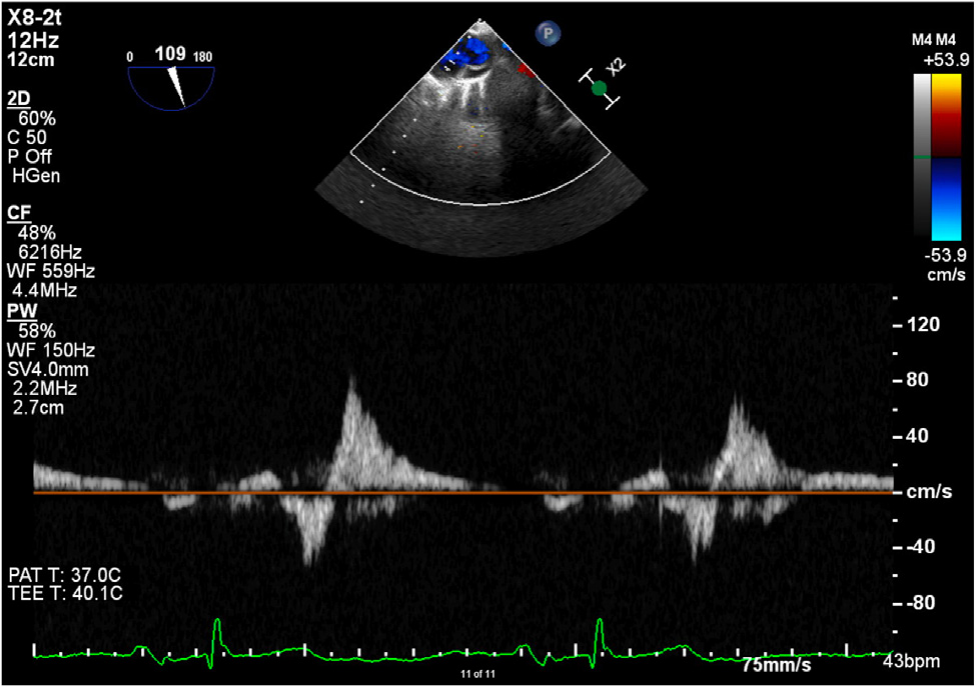
Two-dimensional TEE, midesophageal long-axis (109°) view of the pulmonary veins with color flow Doppler-guided pulsed-wave spectrum, demonstrates systolic flow reversal of the severe MR.

Low–mechanical index (<0.5) TEE demonstrated preserved ejection fraction with severe MR due to posterior leaflet flail, which appeared to involve the P1 scallop and small segment of the lateral commissure on three-dimensional imaging (Figure 4, Video 6). There was a very eccentric anteriorly directed MR jet with systolic flow reversal in the pulmonary veins (Figures 5 and 6, Video 7). The left atrium was severely dilated. Additionally, there was mild aortic regurgitation and TR.

The severe MR was presumed to be chronic in nature given the severely dilated left atrium, moderately increased LV volumes, normal wall motion at the papillary muscle insertion site, and normal LV end-diastolic pressure during angiography. After angiography, the patient denied all cardiac symptoms. As there was no indication for inpatient MV intervention, given the absence of symptoms and borderline LVEF and LV end-systolic diameter on echocardiography, the patient was discharged home in stable condition after appropriate monitoring for anterior ST-segment elevation myocardial infarction. They are currently pending reassessment by their outpatient cardiologist.

## Discussion

MR is the most common valvular abnormality worldwide.^1^ Regurgitant jets are often eccentric in the presence of regional wall motion abnormalities and in the case of leaflet prolapse, flail, or perforation. Noninvasive assessment of eccentric MR using echocardiography can be challenging. This is due to the Coandă effect, the tendency of a fluid jet to follow the trajectory of an adjacent convex surface.^2^ This results in the jet of eccentric MR hugging the left atrial wall, thereby appearing smaller on color flow Doppler in comparison with central jets of the same hemodynamic severity.^3^ Qualitative, spectral density using continuous-wave Doppler may also be unreliable, as a central jet well aligned with the ultrasound beam may appear denser than an eccentric jet of much higher severity, if not well aligned.^4^ For a similar reason, quantitative assessment by flow convergence is challenging given difficulties with poor alignment of the continuous-wave Doppler beam with an eccentric jet. This can lead to an underestimation of jet velocity and an overestimation of the effective regurgitant orifice area.^4^

The use of UEAs is recommended by the American Society of Echocardiography for the quantification of chamber volumes, ejection fraction, and wall motion abnormalities whenever suboptimal images exist.^5^ Furthermore, UEAs are of value in assessment of post–myocardial infarction complications, including pseudoaneurysms and LV apical thrombi.^5^ In terms of valvular assessment, there is abundant literature on the utility of contrast echocardiography for accurate assessment of TR during evaluation of pulmonary hypertension. Given this, the American Society of Echocardiography acknowledges that contrast enhancement of Doppler signals is of value for weak or technically suboptimal TR signals.^5^ Conversely, evaluation of the MV does not hold the same recommendation given the paucity of evidence. Despite this, there appear to be several studies that support the use of contrast echocardiography in MV assessment.

Terasawa *et al.*^6^ evaluated left heart chambers in 31 patients using Doppler echocardiography before and after injection of sonicated albumin contrast. They showed improvement in spectral Doppler velocity profile in all patients with MR. The authors analyzed the velocity profile in detail and concluded that signal improvement was a result of improved uptake in the marginal areas of the Doppler envelope near the cardiac walls where flow velocity was lower and susceptible to removal by cutoff filters as noise. In contrast, at the center of the jet, where flow velocity was high, peak velocity remained unchanged with enhancement.^6^ Unlike the effect of contrast, which increases the detection of peak velocity of maximal TR spectral Doppler profiles, the peak MR spectral Doppler velocity was not significantly altered in this study.^6^^,^^7^ Von Bibra *et al.*^8^ evaluated MR color Doppler signal enhancement in 33 patients using a saccharide-based contrast agent and observed an increase in regurgitant jet area. Of note, the authors noted that in the two patients with eccentric jets from flail mitral leaflet, enhancement remained similar to individuals with central jets.^8^ Despite favorable results, the utility of contrast was not determined, as jet area by color flow Doppler had been shown to have poor correlation with MR severity due to a variety of technical and hemodynamic limitations.^9^

The regular use of UEAs has not been adopted for evaluation of central MR, perhaps because of a failure to improve peak spectral Doppler jet velocity. However, the value of UEAs may be underrecognized in eccentric MR. As seen in our patient and the two patients with flail leaflets in the study of von Bibra *et al.*,^8^ contrast echocardiography was able to overcome the limitation of the Coandă effect, increasing the sensitivity of color flow Doppler detection in eccentric MR. Furthermore, enhanced visualization of the regurgitant jet on color flow Doppler allows better alignment of the ultrasound beam with the eccentric jet, improving spectral Doppler accuracy and overall integrative evaluation of MR severity using 2D transthoracic imaging. Although contrast-enhanced TTE should not be used for quantitative evaluation of MR, given the risk for overestimation in nonholosystolic MR, it may be of value in qualitative assessment of MR, prompting further evaluation with TEE or cardiovascular magnetic resonance imaging.

As demonstrated on our patient’s TEE, commissural lesions can result in very eccentric jets that are difficult to visualize on conventional 2D TTE if the ultrasound beam is not perfectly aligned with the regurgitant jet. Given this, it could be speculated that our findings may be due to misalignment of the ultrasound beam when comparing the nonenhanced and enhanced apical four-chamber windows. Although plausible, the unenhanced two-chamber and three-chamber views did not demonstrate the full extent of the MR jet.

A particular finding that was observed in our patient’s unenhanced 2D TTE was the presence of a distinct horizontal line of color signal across the annular surface of the atrium. This phenomenon was first described by Wiener *et al.*^10^ as color Doppler “splay,” a side-lobe artifact, that was found to be frequently associated with severe MR. This is an additional Doppler sign indicating the need for careful complete evaluation and consideration of TEE.

## Conclusion

We present a patient with MV prolapse with unequivocally severe eccentric regurgitation on TTE that was initially not well visualized on nonenhanced imaging but later highlighted after UEA administration. We suspect that increased echogenicity of the MR jet by UEA increased the sensitivity of color Doppler jet, thereby overcoming the limitation of the Coandă effect. Given this, we would suggest that clinicians consider the use of contrast-enhanced TTE in patients with physical examination findings suggestive of MR who lack such findings on nonenhanced imaging. Furthermore, enhanced color flow Doppler assessment of the MV should be performed on patients receiving UEAs for other conventional indications, as this is a simple and cost-effective method that may help unveil occult MR.

## Ethics Statement

The authors declare that the work described has been carried out in accordance with The Code of Ethics of the World Medical Association (Declaration of Helsinki) for experiments involving humans.

## Consent Statement

Complete written informed consent was obtained from the patient for the publication of this study and accompanying images.

## Funding Statement

This research did not receive any specific grant from funding agencies in the public, commercial, or not-for-profit sectors.

## Disclosure Statement

The authors report no conflict of interest.

## Supplementary Data

Video 1 Nonenhanced 2D TTE, apical four-chamber view, demonstrates the dilated left atrium, borderline LV size with an apical wall motion abnormality, and preserved global LVEF.

Video 2 Nonenhanced 2D TTE, zoomed-in apical four-chamber view, without (*left*) and with (*right*) color flow Doppler, demonstrates the distinct horizontal line of color signal (splay) across the annular surface of the atrium without evidence of the complete regurgitant jet.

Video 3 Nonenhanced 2D TTE, apical three-chamber view, without (*left*) and with (*right*) color flow Doppler, demonstrates the distinct horizontal line of color signal (splay) across the annular surface of the atrium without evidence of the complete regurgitant jet.

Video 4 Nonenhanced 2D TTE, apical two-chamber view, without (*left*) and with (*right*) color flow Doppler, demonstrates the distinct horizontal line of color signal (splay) across the annular surface of the atrium without evidence of the complete regurgitant jet.

Video 5 Two-dimensional TTE, apical four-chamber view, without (*left*) and with (*right*) color flow Doppler after the administration of an UEA, demonstrates the large eccentric anteriorly directed MR jet.

Video 6 Three-dimensional TEE, midesophageal position, volume-rendered display, surgeon’s view of the MV, demonstrates the flail P1 scallop and small flail segment of the lateral commissure.

Video 7 Two-dimensional TEE, midesophageal four-chamber (29°) view, with color flow Doppler, demonstrates the posterior flail leaflet and very eccentric, anteriorly directed MR jet.
